# Supplementary material for: Impacts of an industrial deep-sea mining trial on macrofaunal biodiversity
Source: Nat Ecol Evol. 2025 Dec 5;10(2):318–29. doi: 10.1038/s41559-025-02911-4 (PMC12890587; doi:10.1038/s41559-025-02911-4)
Supplement: Supplementary file 1 — Reporting Summary [file 41559_2025_2911_MOESM1_ESM.pdf]

nature portfolio

Corresponding author(s): Eva Stewart

Last updated by author(s): Aug 28, 2025

Reporting Summary

Nature Portfolio wishes to improve the reproducibility of the work that we publish. This form provides structure for consistency and transparency in reporting. For further information on Nature Portfolio policies, see our Editorial Policies and the Editorial Policy Checklist.

Statistics

For all statistical analyses, confirm that the following items are present in the figure legend, table legend, main text, or Methods section.

n/a	Confirmed	
☐	☒	The exact sample size (*n*) for each experimental group/condition, given as a discrete number and unit of measurement
☐	☒	A statement on whether measurements were taken from distinct samples or whether the same sample was measured repeatedly
☐	☒	The statistical test(s) used AND whether they are one- or two-sided<br>*Only common tests should be described solely by name; describe more complex techniques in the Methods section.*
☒	☐	A description of all covariates tested
☐	☒	A description of any assumptions or corrections, such as tests of normality and adjustment for multiple comparisons
☐	☒	A full description of the statistical parameters including central tendency (e.g. means) or other basic estimates (e.g. regression coefficient) AND variation (e.g. standard deviation) or associated estimates of uncertainty (e.g. confidence intervals)
☐	☒	For null hypothesis testing, the test statistic (e.g. *F*, *t*, *r*) with confidence intervals, effect sizes, degrees of freedom and *P* value noted<br>*Give P values as exact values whenever suitable.*
☒	☐	For Bayesian analysis, information on the choice of priors and Markov chain Monte Carlo settings
☒	☐	For hierarchical and complex designs, identification of the appropriate level for tests and full reporting of outcomes
☒	☐	Estimates of effect sizes (e.g. Cohen's *d*, Pearson's *r*), indicating how they were calculated

Our web collection on statistics for biologists contains articles on many of the points above.

Software and code

Policy information about availability of computer code

Data collection	No software was used to collect data.
Data analysis	R version 4.4.1<br>R package vegan version 2.6-10<br>R package rstatix version 0.7.2<br>R package SpadeR version 0.1.1<br>R package indicspecies version 1.8<br>R package iNEXT version 3.0.1<br>R package BiodiversityR version 2.17-2

For manuscripts utilizing custom algorithms or software that are central to the research but not yet described in published literature, software must be made available to editors and reviewers. We strongly encourage code deposition in a community repository (e.g. GitHub). See the Nature Portfolio guidelines for submitting code & software for further information.

Data

Policy information about availability of data

All manuscripts must include a data availability statement. This statement should provide the following information, where applicable:

- Accession codes, unique identifiers, or web links for publicly available datasets
- A description of any restrictions on data availability
- For clinical datasets or third party data, please ensure that the statement adheres to our policy

All data generated for this study and used in analyses are available in the supplementary materials.

Research involving human participants, their data, or biological material

Policy information about studies with human participants or human data. See also policy information about sex, gender (identity/presentation), and sexual orientation and race, ethnicity and racism.

Reporting on sex and gender	*Use the terms sex (biological attribute) and gender (shaped by social and cultural circumstances) carefully in order to avoid confusing both terms. Indicate if findings apply to only one sex or gender; describe whether sex and gender were considered in study design; whether sex and/or gender was determined based on self-reporting or assigned and methods used. Provide in the source data disaggregated sex and gender data, where this information has been collected, and if consent has been obtained for sharing of individual-level data; provide overall numbers in this Reporting Summary. Please state if this information has not been collected. Report sex- and gender-based analyses where performed, justify reasons for lack of sex- and gender-based analysis.*
Reporting on race, ethnicity, or other socially relevant groupings	*Please specify the socially constructed or socially relevant categorization variable(s) used in your manuscript and explain why they were used. Please note that such variables should not be used as proxies for other socially constructed/relevant variables (for example, race or ethnicity should not be used as a proxy for socioeconomic status). Provide clear definitions of the relevant terms used, how they were provided (by the participants/respondents, the researchers, or third parties), and the method(s) used to classify people into the different categories (e.g. self-report, census or administrative data, social media data, etc.) Please provide details about how you controlled for confounding variables in your analyses.*
Population characteristics	*Describe the covariate-relevant population characteristics of the human research participants (e.g. age, genotypic information, past and current diagnosis and treatment categories). If you filled out the behavioural & social sciences study design questions and have nothing to add here, write "See above."*
Recruitment	*Describe how participants were recruited. Outline any potential self-selection bias or other biases that may be present and how these are likely to impact results.*
Ethics oversight	*Identify the organization(s) that approved the study protocol.*

Note that full information on the approval of the study protocol must also be provided in the manuscript.

Field-specific reporting

Please select the one below that is the best fit for your research. If you are not sure, read the appropriate sections before making your selection.

☐ Life sciences ☐ Behavioural & social sciences ☒ Ecological, evolutionary & environmental sciences

For a reference copy of the document with all sections, see nature.com/documents/nr-reporting-summary-flat.pdf

Ecological, evolutionary & environmental sciences study design

All studies must disclose on these points even when the disclosure is negative.

Study description	This study aims to examine if changes in benthic invertebrate abundance, diversity, and community structure occurred following the test of a commercial prototype polymetallic nodule collector on the abyssal seafloor. To achieve this, seafloor samples were collected using a 50cmx50cm boxcore at 4 sampling times across a two year period. A random stratified sampling design was used, with data collected from four control sites chosen to be spatially equivalent to the area being impacted by the mining disturbance, and one impacted site. A total of 80 quantitative samples were taken, and all individual animals within each sample were identified to a species unit and then used for statistical analysis.
Research sample	Benthic invertebrate macrofauna (>300µm) specimens were obtained from 80 boxcore samples of the abyssal seafloor in the Clarion-Clipperton Zone (CCZ), eastern Pacific Ocean. Individuals were identified by taxonomists to species units, totaling 789 species from a total of 4354 specimens.
Sampling strategy	Sampling procedures followed standard protocols for sampling abyssal macrofauna in the CCZ, see Glover et al. 2015 (https://doi.org/10.3390/jmse4010002). Sampling strategy was based on International Seabed Authority guidelines for establishing

	appropriate environemental baselines for impact assessments, and established protocols for Before-After Control-Impact (BACI) sampling designs.
Data collection	Data were collected on four seagoing expeditions to the CCZ. Samples at sea were collected by ECDS, HW, RD, GBC, CMBB, and MR. Samples were processed in the lab as detailed in the methods by ECDS, HW, CMBB, LK, MBA, GBC, RD, LN, GVD, ASS, and TH.
Timing and spatial scale	The study was conducted in the exploration contract area NORI-D, licenced by the International Seabed Authority (ISA) to Nauru Ocean Resources Inc. The NORI-D contract area is located in the south-eastern CCZ, occupying an area of approximately 27000 km2, and with water depth spanning 2959 – 4602 m. Samples were collected across four expeditions to the NORI-D area spanning a total period of two years. Campaign 5A (C5A) took place from October to November 2020 aboard the Maersk Launcher, and campaign 5D (C5D) took place from April to June 2021 also aboard the Maersk Launcher. Campaigns 7A (C7A), and 7B (C7B) both took place on the MV Island Pride, from August to September 2022 and November to December 2022 respectively. Samples were collected from 5 sampling blocks, each approximately 7.75 km square.
Data exclusions	Data were excluded based on predetermined criteria established for abyssal macrofauna datasets. A macrofauna sensu stricto concept was used when defining the specimens to be included in analyses, this included any metazoan retained on a 300 µm sieve, but excluded traditionally considered meiofaunal taxa such as Ostracoda, Copepoda, Nematoda, and Halicarida, as well as known sessile nodule fauna (the subject of a separate study) and pelagic or likely pelagic contaminants (such as Hyperiid amphipods). Arthropods and annelids were only counted if the head was present, and echinoderms only if the oral disc was present. Sessile animals such as bryozoans (excluding Ctenostomatida which are free-living within the sediment), brachiopods, crinoids, and sponges were separated and sorted, however were not included in subsequent analyses as they are considered nodule fauna (due to their dependence on the substratum) and not sediment fauna. Serpulid polychaetes were excluded from analyses for the same reason, except for certain species which were determined to be free-living.
Reproducibility	The original dataset generated for this study and used to run analyses is provided in the supplementary material. The software and standard code functions are open source and detailed in the methods.
Randomization	Samples were taken from randomised locations within each treatment area.
Blinding	Blinding was not applicable for this study.

Did the study involve field work? ☒ Yes ☐ No

Field work, collection and transport

Field conditions	Near-seabed oceanographic conditions are similar across the CCZ, with typical temperatures = 1.48°C, oxygen = 151µmol/kg, and salinity = 34.87 g.kg-1. Samples were collected at depths from 4121-4312m.
Location	Samples were all collected in the NORI-D contract area in the Clarion-Clipperton Zones, eastern Pacific Ocean. Sites varied in depth from 4121–4312 m
Access & import/export	Samples and data were obtained as part of permitted marine scientific research in areas beyond national jurisdiction (the high seas). The area sampled is licenced for deep-sea mineral exploration to Nauru Ocean Resources Inc. (NORI). NORI holds exploration rights to the NORI-D contract area in the Clarion-Clipperton Zone regulated by the International Seabed Authority and sponsored by the government of Nauru.
Disturbance	Disturbance caused directly by the study was minimal, with all procedures following standard protocols requiring minimal impact on the seabed.

Reporting for specific materials, systems and methods

We require information from authors about some types of materials, experimental systems and methods used in many studies. Here, indicate whether each material, system or method listed is relevant to your study. If you are not sure if a list item applies to your research, read the appropriate section before selecting a response.

Materials & experimental systems

n/a	Involved in the study	
☒	☐	Antibodies
☒	☐	Eukaryotic cell lines
☒	☐	Palaeontology and archaeology
☐	☒	Animals and other organisms
☒	☐	Clinical data
☒	☐	Dual use research of concern
☒	☐	Plants

Methods

n/a	Involved in the study	
☒	☐	ChIP-seq
☒	☐	Flow cytometry
☒	☐	MRI-based neuroimaging

Animals and other research organisms

Policy information about studies involving animals; ARRIVE guidelines recommended for reporting animal research, and Sex and Gender in Research

Laboratory animals	The study did not involve laboratory animals.
Wild animals	The study involved the collection of invertebrate animals in the field (no cephalopods). Animals were collected by boxcore and extracted from the sediment. Organisms recovered to the ship are killed by the temperature and pressure changes between the seafloor and the surface. Organisms collected for this study were small (< 2 cm) and included arthropods, cnidaria, annelids, mollusca, bryozoa, porifera, and echinoderms. The species identified are provided in the supplementary material.
Reporting on sex	N/A.
Field-collected samples	Samples collected from the field were stored in 90% ETOH.
Ethics oversight	No ethical approval or guidance was needed.

Note that full information on the approval of the study protocol must also be provided in the manuscript.

Plants

Seed stocks	*Report on the source of all seed stocks or other plant material used. If applicable, state the seed stock centre and catalogue number. If plant specimens were collected from the field, describe the collection location, date and sampling procedures.*
Novel plant genotypes	*Describe the methods by which all novel plant genotypes were produced. This includes those generated by transgenic approaches, gene editing, chemical/radiation-based mutagenesis and hybridization. For transgenic lines, describe the transformation method, the number of independent lines analyzed and the generation upon which experiments were performed. For gene-edited lines, describe the editor used, the endogenous sequence targeted for editing, the targeting guide RNA sequence (if applicable) and how the editor was applied.*
Authentication	*Describe any authentication procedures for each seed stock used or novel genotype generated. Describe any experiments used to assess the effect of a mutation and, where applicable, how potential secondary effects (e.g. second site T-DNA insertions, mosiacism, off-target gene editing) were examined.*
